# Fast Gelation of Poly(ionic liquid)-Based Injectable Antibacterial Hydrogels

**DOI:** 10.3390/gels8010052

**Published:** 2022-01-12

**Authors:** Che Zhao, Chengju Sheng, Chao Zhou

**Affiliations:** 1 School of Aerospace and Mechanical Engineering, Changzhou Institute of Technology, Changzhou 213032, China; zhaoche@czu.cn; 2 School of Chemistry and Chemical Engineering, Southwest University, Chongqing 400715, China; 3 Institute of Biomedical Engineering and Health Sciences, Changzhou University, Changzhou 213164, China

**Keywords:** antibacterial agents, imidazolium poly(ionic liquids), injectable hydrogels, anti-inflammatory

## Abstract

Traditional antibacterial hydrogels have a broad-spectrum bactericidal effect and are widely used as wound dressings. However, the biological toxicity and drug resistance of these antibacterial hydrogels cannot meet the requirements of long-term clinical application. Imidazolium poly(ionic liquids) (PILs) are polymeric antibacterial agents exhibiting strong antibacterial properties, as they contain a strong positive charge. In this study, two imidazolium PILs, namely poly(*N*-butylimidazolium propiolic acid sodium) (PBP) and poly(*N*-(3,6-dioxaoctane) imidazolium propiolic acid sodium) (PDP), as high efficiency antibacterial agents, were synthesized by polycondensation reaction. Then, the PILs were compounded with polyethylene glycol (PEG) by a thiol-yne click reaction to prepare injectable antibacterial hydrogels. An in vitro assay showed that the injectable antibacterial hydrogels could not only quickly kill *Escherichia coli* (*E. coli*) and *Staphylococcus aureus* (*S. aureus*), but also had low toxicity for human skin fibroblasts cells (HSFs) and human umbilical vein endothelial cells (HUVECs), respectively. Additionally, the lipopolysaccharide (LPS) inflammation model revealed that the injectable antibacterial hydrogels also had anti-inflammatory effects, which would be advantageous to accelerate wound healing.

## 1. Introduction

Injectable hydrogels have great application prospects in tissue engineering, because they have good water absorption, air permeability, and biocompatibility [1,2]. Injectable hydrogels can precisely deliver cells or drugs without invasive surgery, thus reducing the risk of infection, minimizing trauma to the surrounding tissue and organs, and alleviating pain [3,4]. Specifically, injectable hydrogels containing antibacterial agents as implanted medical devices represent a conventional strategy to reduce infection [5]. Traditional injectable antibacterial hydrogels contain silver nanoparticles [6], antimicrobial peptides [7], antibiotics [8], chitosan [9], or graphene oxide [10], but these antibacterial hydrogels have certain biological toxicity and drug resistance, which cannot meet the requirements of long-term clinical application [11,12].

Poly(ionic liquids) (PILs) consists of repeating units of polymer backbone and ionic liquid (IL) species [13,14]. Recently, various imidazolium PILs have been synthesized and exhibited high sterilization rates against *E. coli* and *S. aureus*, as well as multi-drug resistant bacteria, such as methicillin-resistant *Staphylococcus aureus* (MRSA) [15,16,17,18,19,20]. The imidazolium PILs can serve as the antibacterial agents, and they are gaining more and more attention. The antibacterial mechanism of PILs involves positive imidazolium groups in the PILs attracting the negatively charged bacterial cell wall under the action of electrostatic force. Then, the hydrophobic segment in the PILs penetrates the cell wall and cell membrane of bacteria [21,22,23].

Most reported imidazolium PILs contain chloride, bromide, iodide, zinc, and copperas as counter ions, which could pose a hazard to normal cells [15,16,18,19,24]. Furthermore, few papers reported antibacterial hydrogels with PILs. Recently, our research focused on preparing less toxic carboxyl groups as anions in imidazolium PILs and fabricating highly effective antibacterial hydrogels with the PILs [20]. However, the antibacterial hydrogels containing PILs showed slow gelation and could not be injectable, which limited the application as wound dressing in clinical practice. In this work, we fabricated fast gelation and injectable antibacterial hydrogels, which would be well adaptable to cover any irregularly infected wound completely in future clinical situations.

**Figure 1:**
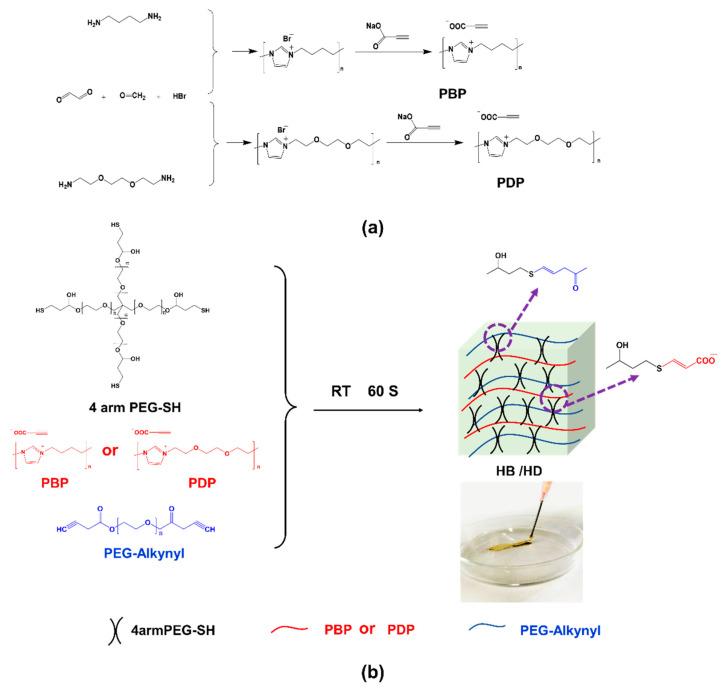
Schematic presentation for synthesis of (**a**) PBP and PDP and (**b**) injectable hydrogels.

Firstly, two antibacterial imidazolium PILs, namely poly(*N*-butylimidazolium propiolic acid (PBP) and poly(*N*-(3,6-dioxaoctane) imidazolium propiolic acid) (PDP)), were obtained via polycondensation reaction (Figure 1a) [25]. The synthesized imidazolium PILs containing alkynyl and biocompatible poly(ethylene glycol) (PEG) propiolate (PEG-Alkynyl) were crosslinked with 4 arm thiol PEG (4 arm PEG-SH) to fabricate fast gelation and injectable antibacterial hydrogels by thiol-yne click reaction (Figure 1b). The 4 arm PEG-SH played the role of the backbone here, combining imidazolium PILs and PEG-Alkynyl, and the molecular weight was about 20,000, which demonstrated good crosslinking efficiency [26].

Finally, physical properties antibacterial and anti-inflammatory properties of the injectable antibacterial hydrogels for in vitro assays were evaluated.

## 2. Results and Discussion

### 2.1. Structure of Synthesized PILs

PBP and PDP were synthesized by polycondensation reaction, followed by an alkynyl counter ion exchange reaction. ^1^H nuclear magnetic resonance (^1^H NMR) of both PBP and PDP (Figure S1) showed chemical shifts at 4.25 ppm, which corresponded to the methylene protons connecting to the quaternary nitrogen atoms [17]. The integration ratio of the alkynyl at 1.75 ppm and the imidazolium ring at 7.48 ppm was 1:2. The Fourier transform infrared spectroscopy (FTIR) spectra in Figure S3a show that vibration peaks at 2200 cm^−1^ and 1700 cm^−1^ corresponded to –C≡C– and C=O, respectively, in both PBP and PDP. In addition, there was a vibration peak at 1200 cm^−1^ that corresponded to the C–O of PDP. These data confirmed that the targeted PILs (PBP and PDP) were successfully synthesized. The zeta potentials of PBP and PDP were 6.9 mV and 8.5 mV (Figure S3b), respectively, which were attributed to the cationic imidazolium groups in PILs. The particle sizes of PBP and PDP were 128 nm and 148 nm, respectively, characterized by dynamic light scattering (DLS), as shown in Figure S3c.

### 2.2. The Gelation and Physical Properties of Injectable Hydrogels

The as prepared PILs contained propiolic acid as anionic group and was mixed with PEG-Alkynyl in Dulbecco’s phosphate buffered saline (PBS) solute on 4 arm PEG-SH as the crosslinker was added to the solution. Finally, the injectable hydrogels were fabricated by thiol-yne click reaction. The injectable hydrogels prepared from PBP were named as HB and prepared from PDP were named as HD. As controls, hydrogels without PILs were named as HN.

**Figure 2:**
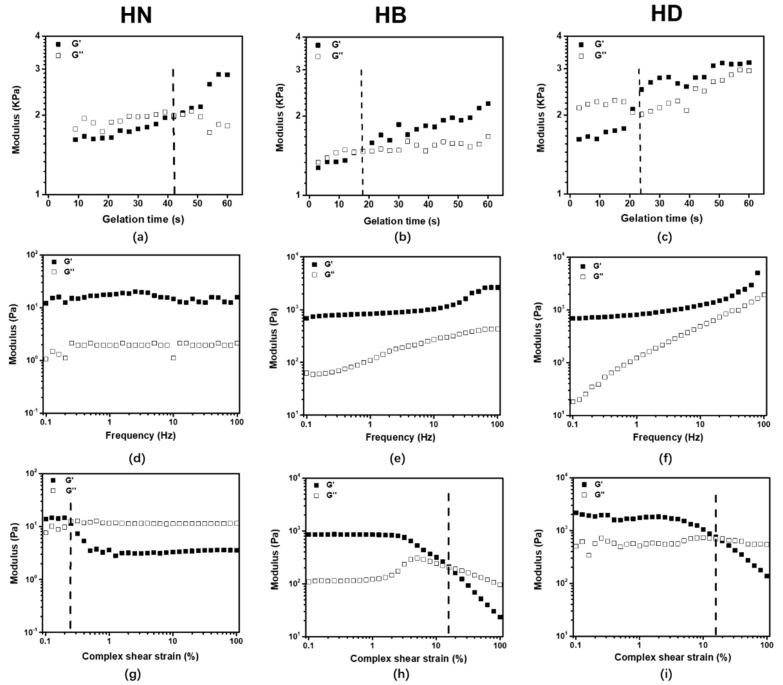
The gelation time (**a**–**c**) at a constant frequency (*f* = 1 Hz); frequency sweep (**d**–**f**) with constant oscillatory strain (*γ* = 0.5%) and *f* = 0.1–100 Hz; amplitude sweep (**g**–**i**) with *f* = 1 Hz and *γ* = 0.1–100%.

A suitable injectable hydrogel should be easily injected and rapidly crosslinked [27,28]. The gelation time of hydrogels was determined by oscillatory rheometry. The results depicted in Figure 2a–c show that the modulus of all hydrogels was increased and the gelation time of hydrogels was less than 60 s. The gelation time of HB (15 s) and HD (20 s) were shorter than that of HN (40 s). On the one hand, a thiol-yne click reaction occurred between alkynyl groups and thiol groups and the viscosity increased to form hydrogels [29,30,31]. On the other hand, the PBP and PDP penetrated PEG hydrogels and could provide more alkynyl groups and accelerate the thiol-yne click reaction [32,33]. From the gelation time results, it was certified that all hydrogels prepared by thiol-yne were injectable (Figure 1).

In order to characterize the viscoelastic behaviors of the injectable hydrogels, the hydrogels were measured by oscillatory rheometry. The frequency sweep results showed that storage modulus (G′) of all hydrogels was significantly higher than loss modulus (G″), indicating that the hydrogels presented elastic property. Moreover, G′ and G″ of HB and HD were gradually increased with frequency, and G′ of HB and HD were larger than that of HN. This is because PILs could act as a crosslinker in the hydrogels (Figure 2d–f). The amplitude sweep results showed that HN was fractured at 0.2–0.3% strains, suggesting that this hydrogel was brittle (Figure 2g–i). In contrast, HB and HD were all fractured at around 19% strains. That was because that PILs could penetrate injectable hydrogels to form a semi-interpenetrate (semi-IPN) network, then the crosslinking density of hydrogels increased [34,35].

Table S1 indicates the swelling ratio of freeze-dried hydrogels calculated by Equation (1). The HN hydrogels showed a higher swelling ratio, reaching 754%. On the contrary, under the same conditions, the swelling ratio of HB and HD was lower (421% and 373%, respectively). The crosslinking density of injectable hydrogels was calculated by Equations (2) and (3), and the result showed that the crosslinking density of HB (0.41 mol/cm^3^) and HD (0.52 mol/cm^3^) was higher than that of HN (0.13 mol/cm^3^), as shown in Table S1. This was because PILs in HB and HD could increase the crosslinking density of hydrogels [36,37]. Moreover, according to the field emission scanning electron microscope (FE-SEM) images (Figure S4), the pore size of HN, HB, and HD were 32 μm, 24 μm, and 13 μm, respectively, which also demonstrates that the crosslinking density of the hydrogels increased with the decrease of the pore size.

**Figure 3:**
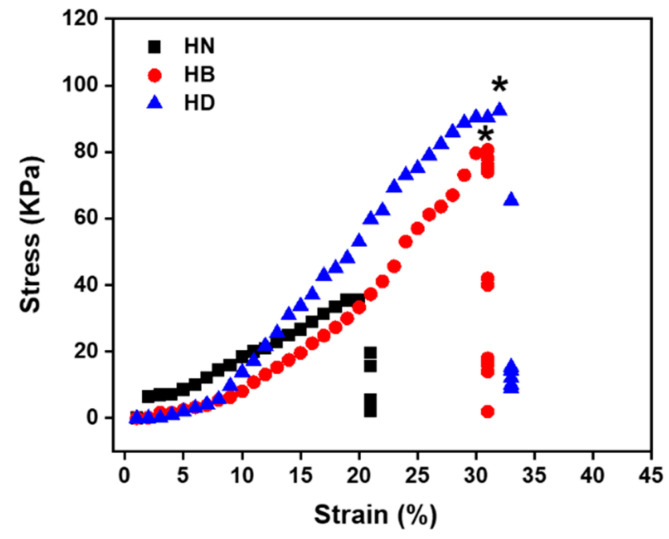
Stress–strain curves for the compressive testing at 25 °C of HN, HB and HD after freeze-drying (*n* = 4, * means *p* ≤ 0.5).

The uniaxial compression tests were performed on freeze-dried hydrogels to evaluate their mechanical properties. All the hydrogels displayed a rupture compression stress of HN, HB, and HD, which were 37.6 kPa 80.8 kPa, and 95.4 kPa, respectively (Figure 3). The results revealed that PILs penetrated into HN hydrogels and improved their mechanical property.

### 2.3. In Vitro Antibacterial and Antibiofilm Activities of Injectable Hydrogels

**Figure 4:**
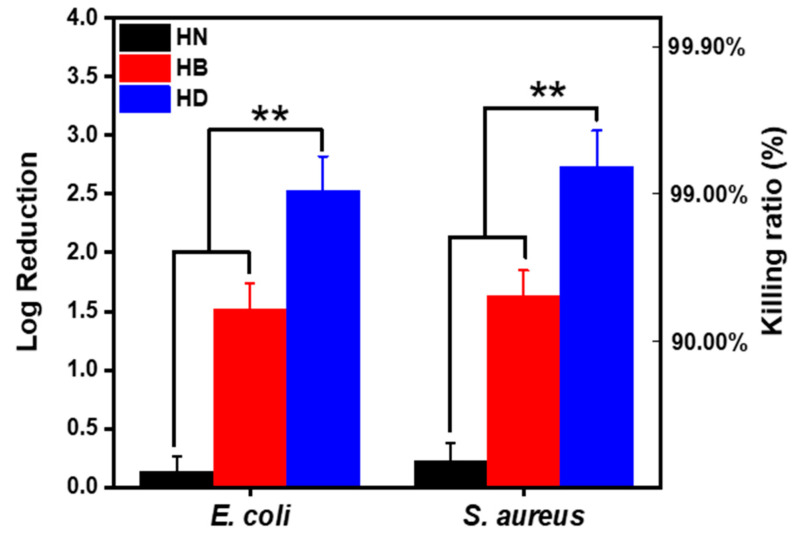
Injectable hydrogels surface colonies of *E. coli* and *S. aureus* after 2 h in vitro (PBS as control) (*n* = 4, ** means *p* ≤ 0.01).

The antibacterial activities of the prepared injectable hydrogels (HB and HD) containing imidazolium PILs are shown in Figure 4. Both HB and HD exhibited antibacterial activities against *E. coli* and *S. aureus*. HD could reduce the amount of *E. coli* by a 2.51 log reduction (sterilization rate = 99.5%) and *S. aureus* by a 2.64 log reduction (sterilization rate = 99.7%), in both cases more effectively than that of HB (reducing the amount of *E. coli* by a 1.5 log reduction for (sterilization rate = 95.5%) and *S. aureus* by a 1.6 log reduction (sterilization rate = 96.4%)). That was because the zeta potential of PDP (8.5 mV) was higher than that of PBP (6.9 mV). The more positive charge imidazolium groups on the polymer chains, the better disruption of the bacteria cell wall [38]. This result was also consistent with minimum inhibitory concentrations (MICs) of HB and HD in Table S2.

**Figure 5:**
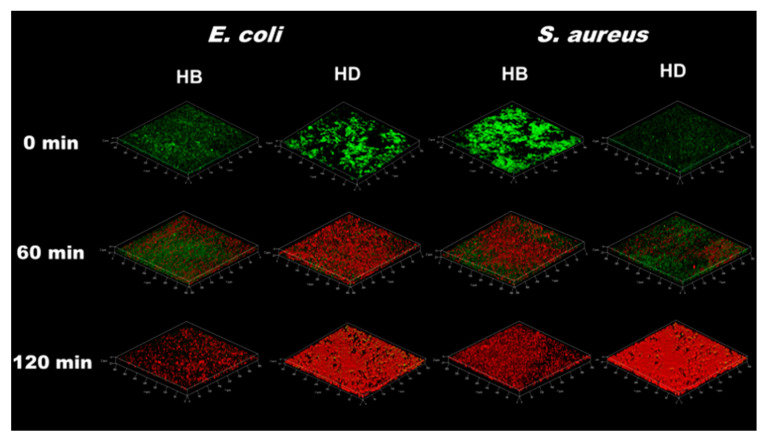
Fluorescence images of biofilms stained with LIVE/DEAD stain after treating with HB and HD in 0 min, 60 min and 120 min.

**Figure 6:**
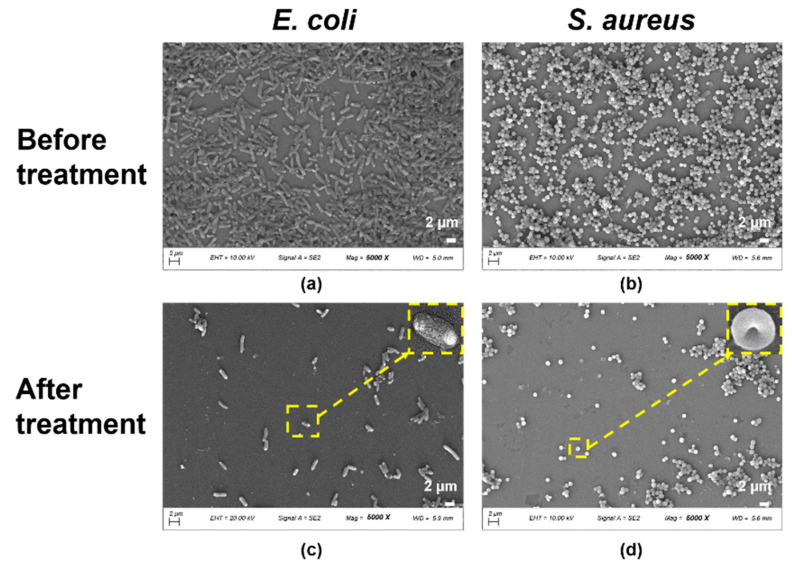
FE-SEM images of (**a**) *E. coli* and (**b**) *S. aureus* seeded for 24 h, (**c**,**d**) are images after treatment with hydrogel HD (The yellow frames represented the dead bacteria after hydrogel treatment and the scale bars are 2 μm).

In order to investigate the antibiofilm property of injectable hydrogels, the Live/Dead biofilm *E. coli* and *S. aureus* was characterized by a contacted killing model with the injectable hydrogels containing PILs. It could be clearly observed that most of the dead bacteria appeared after HB was contacted to the biofilm for 2 h by green/red-fluorescent-labeled in Figure 5. While most bacteria turned red only for 1 h after HD exposure to biofilm. The result was consistent with in vitro antibacterial activity. Additionally, it illustrated that the biofilms of *E. coli* and *S. aureus* were destroyed, and a large amount of bacterial lysis and death occurred with HD treatment over 2 h (Figure 6). The mechanism of antibiofilm actives involved the electrostatic interactions between cationic moieties of PILs and the bacterial cell membranes, and then the hydrophobic carbon segments of PILs penetrated the phospholipid bilayers of the bacterial cell membranes [39].

### 2.4. In Vitro Cytotoxicity of Injectable Hydrogels

**Figure 7:**
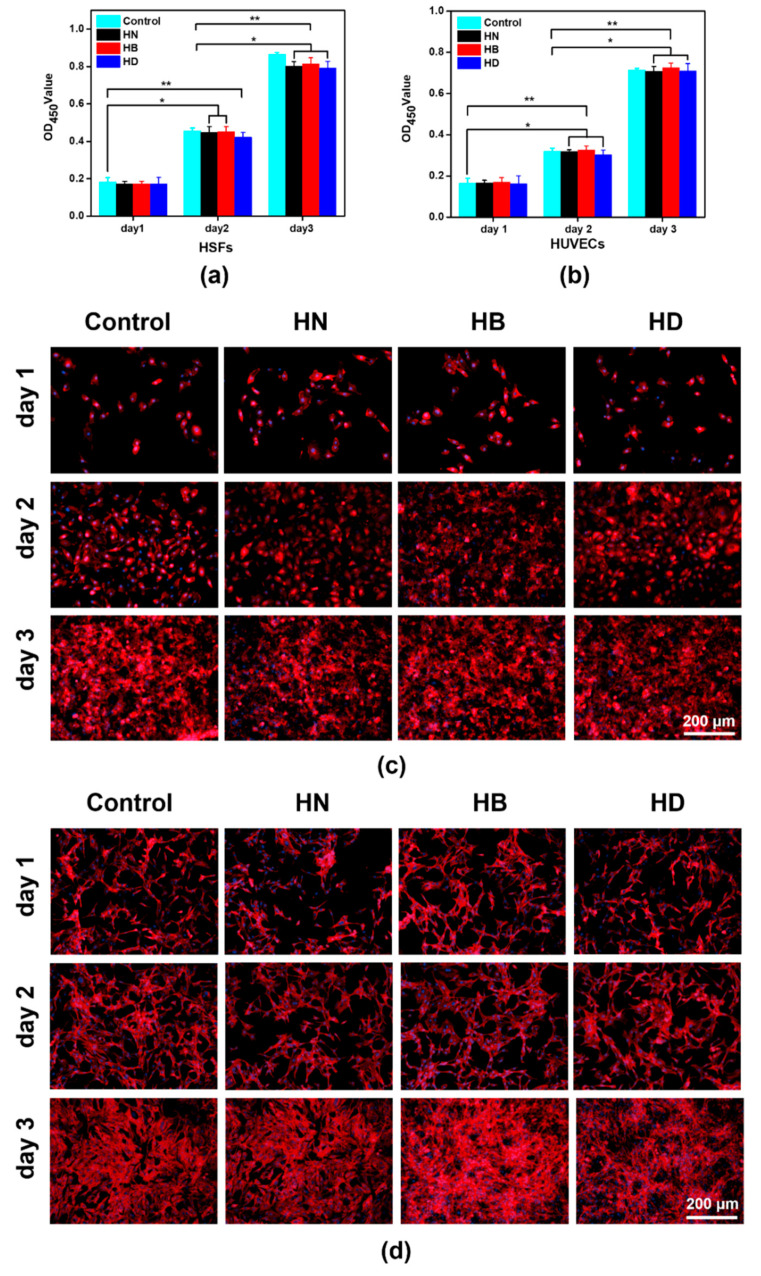
Cell viability and morphology when cultured on injectable hydrogels: (**a**) cell viability of HUVECs at day 1, day 2 and day 3; (**b**) cell viability of HSFs at day 1, day 2 and day 3; (**c**) cell morphology of HUVECs at day 1, day 2 and day 3; (**d**) cell morphology of HSFs at day 1, day 2 and day 3; the scale bars are 200 μm (*n* = 4, * means *p* ≤ 0.5, ** means *p* ≤ 0.01).

The cytotoxicity of injectable antibacterial hydrogels is also one of the important indicators for clinical application [40,41]. In this work, HUVECs and HSFs were used to evaluate the cytotoxicity of injectable antibacterial hydrogels, because they had an important influence on skin morphogenesis in reconstructed human skin equivalents and vascular repair [42,43]. A cell counting Kit-8 (CCK-8) method was used to quantify the cytotoxicity of the hydrogels at day 1, day 2, and day 3, respectively. The OD values of the injectable hydrogels were increased in three days (Figure 7a,b), which certified that the hydrogels containing PILs had less cytotoxicity for HUVECs and HSFs, respectively. According to cell morphologies of HUVECs and HSFs treated with hydrogel extracellular matrix shown in Figure 7c,d, the number of cells was increased in three days. The results were in good agreement with OD values. Furthermore, a great number of filopodia of HUVECs and HSFs appeared at day 3, which also illustrated that the injectable hydrogels containing PILs could not elicit cytotoxicity [44].

### 2.5. In Vitro Hemolytic Activity and Anti-Inflammatory Activity of Injectable Hydrogels

Hemolysis was used to study the hemocompatibility of the injectable hydrogels, because the hydrogels could cover the injury area when it served as a barrier device for adhesion prevention [45]. According to the results, all hydrogels were below 0.7% in hemolytic activities (Figure S5), indicating that these hydrogels are considered non-hemolytic biomaterials, which was consistent with American Society for Testing and Materials (ASTM) F756-00 standard [46].

**Figure 8:**
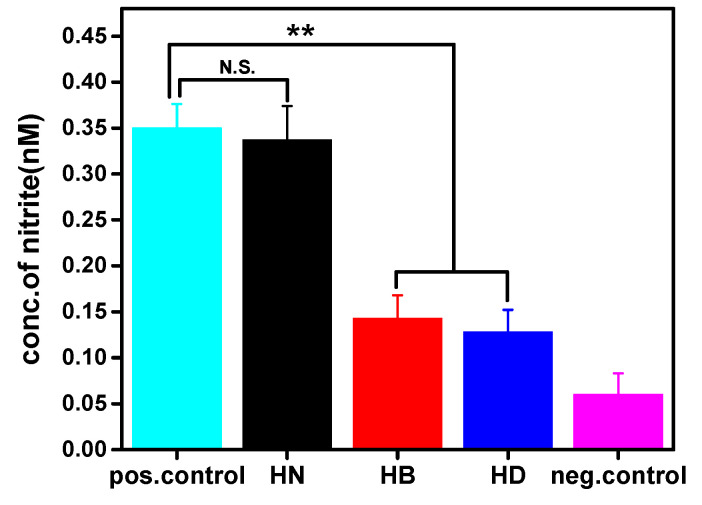
Anti-inflammatory activity of U-937 cells treated with LPS alone (positive control) and injectable hydrogels, and cells without any treatment (negative control) as measured by a Griess assay (*n* = 4, N.S. means no significant difference, ** means *p* ≤ 0.01).

According to the results of Griess assay (Figure 8), HB and HD had ability to suppress nitric oxide (NO) production activated by LPS. This demonstrated that PILs in the hydrogels had therapeutic efficacy. This is because LPS is a component of the outer cell wall of Gram-negative bacteria and PILs could kill *E. coli* effectively, thereby reducing the production of NO in U-937 cells induced by LPS [47]. More importantly, through the LPS inflammation model [48,49], we observed that the prepared injectable hydrogels containing PILs could quickly destroy their antibiofilm activity within 2 h, thereby preventing the inflammatory reaction to a certain extent.

## 3. Conclusions

In summary, imidazolium PILs (PBP and PDP) were synthesized by polycondensation reaction and characterized by ^1^H NMR, FT-IR, DLS, and electrophoretic light scattering. The injectable hydrogels (HB and HD) had higher mechanical strength and shorter gelation time compared with HN. Moreover, HD could be better used in the field of antibacterial dressings due to its higher molecular weight and better hydrophilicity [50]. In vitro assays showed that these injectable hydrogels could kill *E. coli* and *S. aureus* with a killing rate of more than 95% within 2 h, and HD could reach 99%. They also had low hemolytic activities and exhibited anti-inflammatory property. Unlike the existing injectable antibacterial hydrogels, our injectable hydrogels not only formed quickly, but also killed bacteria effectively and demonstrated good anti-inflammatory property. Moreover, we consider a wide range of applications in the field of biomedicine.

## 4. Materials and Methods

### 4.1. Materials

The 4 arm PEG (*M*_n_ = 20,000) was purchased from SinoPEG, Xiamen, China. PEG, (*M*_n_ = 2000) was purchased from Alfa Aesar, Shanghai, China. Dichloromethane (99%), diethyl ether (99%), 1,4-diaminobutane (98%), 1,2-bis(2-aminoethoxy)ethane (98%), hydrobromic acid (48 wt.% in H_2_O), and 3-mercaptopropionic acid (98%) were purchased from Aladdin, Shanghai, China. Dichloromethane (CH_2_Cl_2_, 99.8%), sulfuric acid (H_2_SO_4_, 99.9%), toluene (99.5%), PBS, propiolic acid (95%), glyoxal (40%), formaldehyde (40%), LPS, dehydrated alcohol (CH_3_CH_2_OH), and Roswell Park Memorial Institute (RPMI-1640) medium were purchased from Sigma-Aldrich, St. Louis MO, USA. Propiolic acid sodium salt (99%) was purchased from J&K Scientific, Beijing, China. Dulbecco’s modified eagle’s medium-high glucose (DMEM-H), Endothelial culture medium (ECM), fetal bovine serum (FBS), L13152 LIVE/DEAD^®^ Bac Light TM Bacterial Viability, Kit phalloidin-TRITC and 4′,6-diamidino-2-phenylindole (DAPI) were purchased from Thermo Fisher Scientific, Waltham, MA USA. *E. coli* (DH 5α), *S. aureus* (ATCC 25923), HUVECs, HSFs, U-937 cells, lysogeny broth (LB), Mueller-Hinton broth (MHB), Mueller-Hinton agar (MHA), and CCK-8 were purchased from BeyoClick™, Shanghai, China. Triton X-100 (0.3%) was purchased from SAIES, Nanjing, China, while 4% paraformaldehyde was purchased from Boster Biological Technology Co., Wuhan, China. The Griess reagent kit (ab234044) was purchased from Sigma-Aldrich, St. Louis, MO, USA.

### 4.2. Synthesis of PEG-SH and PEG-Alkynyl

The synthesis of 4 arm PEG-SH was performed following a published method [51]. The 4 arm PEG and mercaptopropionic acid were dissolved in toluene and two drops of H_2_SO_4_ were added into the solution at 80 °C. The resultant viscous liquid was dissolved in dichloromethane and washed with saturated NaHCO_3_ solution. The organic phase was dried using MgSO_4_, then filtered and evaporated in vacuo to yield the product as a slight yellow oil.

^1^H NMR (500 MHz, CDCl_3_, d, δ (ppm): 1.70 (s, S-*H*), 2.66 (s, O=C–C*H*_2_), 2.85 (d, C*H*_2_–SH), 3.57–3.70 (m, C*H*_2_–C*H*_2_–O), 4.26 (s, O–C*H*_2_)). The synthesis of PEG-Alkynyl was similar to the above procedure and yielded the product as a white powder (15.3 g, 75%). ^1^H NMR (500 MHz, CDCl_3_, d, δ (ppm): 2.12 (s, CH_2_–C*H*_2_–CH_2_), 3.01 (s, C≡C*H*), 3.57–3.70 (m, C*H*_2_–C*H*_2_–O), 4.26 (s, C*H*_2_–C=O)).

### 4.3. Synthesis of PBP and PDP

PILs containing a counter ion bromide, poly(N-butylimidazolium bromide), and poly(*N*-(3,6-dioxaoctane) imidazolium bromide were prepared according to a previously published method [25]. Three equivalents of propiolic acid sodium salt were mixed one equivalent of 1,4-diaminobutane or 1,2-bis(2-aminoethoxy)ethane to exchange with bromide. The products were dialyzed for 24 h (MWCO = 1000 Da) to remove byproducts. The as prepared PILs salts (PBP and PDP) were composed of imidazolium as cation and propiolic carboxylate as anion. After lyophilization, PBP and PDP were obtained each with a yield of 80%. PBP (^1^H NMR, 500 MHz, D_2_O, δ (ppm): 8.3 (d, –N–C*H*=CN^+^), 7.48 (d, N–C*H*=C*H*–N^+^), 4.25 (d, N^+^–C*H*_2_–C*H*_2_–CH_2_–CH_2_), 1.74 (s, COOH–C≡C*H*), 1.75 (m, N^+^–CH_2_–CH_2_–C*H*_2_–C*H*_2_)). PDP (^1^H NMR, 500 MHz, D_2_O, δ (ppm): 8.3 (d, –N–C*H*=CN^+^), 7.48 (d, N–CH=CH–N^+^), 4.25 (dd, N^+^–C*H*_2_–C*H*_2_-O), 3.55–3.75 (m, CH_2_–O–C*H*_2_–C*H*_2_–O–C*H*_2_–C*H*_2_) and 1.74 (s, COOH–C≡C*H*)).

### 4.4. Preparation of Antibacterial Hydrogels

According to various previous measurements with respect to PILs, antibacterial hydrogels were prepared from PILs (PBP and PDP), 4 arm PEG-SH, and PEG-Alkynyl. Four-arm PEG-SH (0.1 g/mL), PEG-Alkynyl (0.025 g/mL), and PILs (PBP or PDP each to 0.005 g/mL) were first dissolved in 200 μL of PBS solution, and the reaction was allowed to occur in ampoule at room temperature for 60 s. Prior to use, all hydrogels were sterilized by filtration (0.22 μm filter, Millipore, Burlington, MA, USA).

### 4.5. Characterization of PILs and Hydrogels

^1^H NMR were recorded with a 500 MHz Bruker Avance instrument and FTIR were recorded with Nicolet-460 (Thermo Fisher, Waltham, MA, USA). The diameters and zeta potentials of PILs were determined by DLS at 25 °C and electrophoretic light scattering (ELS) using a zeta Sizer Nano ZS (Malvern Instruments, Westborough, MA, USA).

The gelation time and the rheological properties of the hydrogels were evaluated at a constant frequency *f* = 1 Hz (25 °C) by a Malvern Kinexus pro rheometer. Following hydrogel formation, the frequency sweep experiment was performed at *γ* = 0.5%, and *f* = 0.1–100 Hz, and the amplitude sweep experiment was performed *f* = 1 Hz and *γ* = 0.1–100%.

The surface morphology of the freeze-dried hydrogels was observed using FE-SEM (Zeiss Sigma 500, Oberkochen, Germany). All samples were coated with gold before characterization.

Compressive stress–strain measurements of the freeze-dried hydrogels (*d* = 2 mm, *h* = 5 mm) were obtained using compression test machine (Bose ELF3200, Framingham, MA, USA). The load cell was 10 N, and the compression velocity was 1 mm/min. The stress and strain values were taken at the rupture point when the freeze-dried hydrogels brake.

### 4.6. Equilibrium Swelling and Crosslinking Density of Injectable Hydrogels

The swelling ratio Q and crosslinking density $\rho_{s}$ were calculated from the following Equations (1) and (2) [26].

(1) $$Q=\frac{W_{d}-W_{s}}{W_{s}}\times 100\%$$

where $W_{s}$ and $W_{d}$ are the weights of the freeze-dried hydrogel and fully swollen hydrogel, respectively [52].

The equilibrium polymer volume fraction, $V_{2}$, which was the ratio of the dry gel volume and swollen gel volume, could be related to the swelling ratio Q following Equation (2).

(2) $$V_{2}=\frac{\rho_{s}}{{Q\;\rho }_{p}+\rho_{s}}$$

where $\rho_{s}$ is the density of swelling media, which was water, and $\rho_{p}$ the density of the freeze-dried hydrogel.

### 4.7. Antibacterial Assay and Cytocompatibility of PILs

MICs of *E. coli* and *S. aureus* of PILs were determined using a method previously reported [11]. The cytocompatibility evaluation of the PILs was qualified by the CCK-8 method, and a detailed procedure followed a previously published report [53].

### 4.8. In Vitro Antibacterial and Antibiofilm Assay for Hydrogels

The procedure of antibacterial assay followed a published method [54]. First, the hydrogels were completely formed in the 48-well microplate at 25 °C. The hydrogels in the plates were soaked and rinsed with sterilized PBS overnight, and the PBS was refreshed every 5 h. Hence, 10 μL of bacterial suspension in sterilized PBS (10^6^ CFU/mL of *E. coli* or 10^7^ CFU/mL of *S. aureus*) was spread onto each hydrogel surface in 48-well culture plate, which were then incubated at 37 °C for 2 h. Then 1 mL sterilized PBS was added to each well to re-suspend any bacterial survivors. The CFU was determined through the serial dilution method. Tests were repeated three times for each group and the log reduction value was calculated by Equation (3).

(3) $${\mathrm{Log}\;\mathrm{reduction}\;\mathrm{value}=\log}_{10}[\frac{{\mathrm{CFU}}_{\mathrm{control}}}{{\mathrm{CFU}}_{\mathrm{sample}}}]$$

where CFU_control_ is the CFU of PBS and CFU_sample_ is the CFU of HN, HB, and HD.

The antibiofilm activities with respect to *E. coli* and *S. aureus* of the hydrogels were evaluated according to a previous report [34]. The injectable hydrogels covered the biofilm which formed from bacterial suspensions for 120 min. Subsequently, the fluorescence microscope and FE-SEM were used to observe biofilms after the hydrogels were removed.

### 4.9. Cytocompatibility Evaluation of the Hydrogels

A CCK-8 method was applied to investigate HUVECs and HSFs viability by quantifying the live and dead cells cultured with the hydrogels. The procedure of cytocompatibility evaluation of hydrogels followed a published method [54]. The absorbed peak was measured using a microplate reader (Infinite F50) at a wavelength of 450 nm.

Cell viability was calculated by Equation (4).

(4) $$\mathrm{Cell}\;\mathrm{viability}\;\%=\frac{{\mathrm{OD}}_{\mathrm{sample}}}{{\mathrm{OD}}_{\mathrm{control}}}\times 100\%$$

### 4.10. In Vitro Hemolytic Assay and Anti-Inflammatory Activity of Hydrogels

The procedure for the hemolytic assay of hydrogels followed a published method [34]. The hemolysis percentage was calculated by Equation (5).

(5) $$\mathrm{Hemolysis}\%=\frac{{\mathrm{OD}}_{\mathrm{sample}}-{\mathrm{OD}}_{\mathrm{negative}}}{{\mathrm{OD}}_{\mathrm{positive}}-{\mathrm{OD}}_{\mathrm{negative}}}\times 100\%$$

The procedure for evaluating the in vitro anti-inflammatory activity of the hydrogels followed a published method. The anti-inflammatory activity was determined by the amount of nitric oxide (NO) released in the form of nitrite using the Griess assay [34,55].

### 4.11. Statistical Analysis

All data are presented as the mean ± standard deviation (SD). Statistical significance was determined by Tukey’s post hoc analysis (SPSS statistics 23, IBM Inc., Armonk, NY, USA). * *p* ≤ 0.05, ** *p* ≤ 0.01, *** *p* ≤ 0.001 were considered statistically significant.

## Data Availability

Not applicable.

## Supplementary Materials

The following supporting information can be downloaded at: https://www.mdpi.com/article/10.3390/gels8010052/s1, Figure S1: ^1^H NMR (500 MHz, D_2_O) of (A) PBP and (B) PDP; Figure S2: ^1^H NMR (500 MHz, CDCl_3_) of (A) 4 arm PEG-SH and (B) PEG-Alkynyl; Figure S3: Characterization of poly(ionic liquids) (a) FTIR of PBP and PDP; (b) zeta potential of PBP and PDP and (c) nano particle size of PBP and PDP; Figure S4: Surface morphology of (a) HN; (b) HB; (c) HD by FE-SEM; Figure S5: Hemolysis of hydrogels; Table S1: Swelling characteristics and crosslinking density of injectable hydrogels in PBS (pH = 7.4) at 37 °C; Table S2: Antibacterial and cell viability of PBP and PDP.
