# Supplementary material for: Fast Gelation of Poly(ionic liquid)-Based Injectable Antibacterial Hydrogels
Source: Gels. 2022 Jan 12;8(1):52. doi: 10.3390/gels8010052 (PMC8775204; doi:10.3390/gels8010052)
Supplement: Supplementary file 1 [file gels-08-00052-s001.zip › gels-1529225-supplementary.pdf]

gels

MDPI

Supplementary

Fast Gelation of Poly(ionic liquid)-Based Injectable Antibacterial Hydrogels

Che Zhao [1], Chengju Sheng [2,*] and Chao Zhou [3,*]

[1] School of Aerospace and Mechanical Engineering, Changzhou Institute of Technology, Changzhou 213032, China; zhaoche@czu.edu.cn
[2] School of Chemistry and Chemical Engineering, Southwest University, Chongqing 400715, China
[3] Institute of Biomedical Engineering and Health Sciences, Changzhou University, Changzhou 213164, China
* Correspondence: scj1995@email.swu.edu.cn (C.S.); zhouchao@cczu.edu.cn (C.Z.)

Citation: Zhao, C.; Sheng, C.; Zhou, C. Fast Gelation of Poly(ionic liquid)-Based Injectable Antibacterial Hydrogels. *Gels* **2022**, *8*, 52. https://doi.org/10.3390/gels8010052

Academic Editor: Rama Bansil

Received: 13 December 2021
Accepted: 9 January 2022
Published: 12 January 2022

Publisher's Note: MDPI stays neutral with regard to jurisdictional claims in published maps and institutional affiliations.

Copyright: © 2022 by the authors. Submitted for possible open access publication under the terms and conditions of the Creative Commons Attribution (CC BY) license (https://creativecommons.org/licenses/by/4.0/).

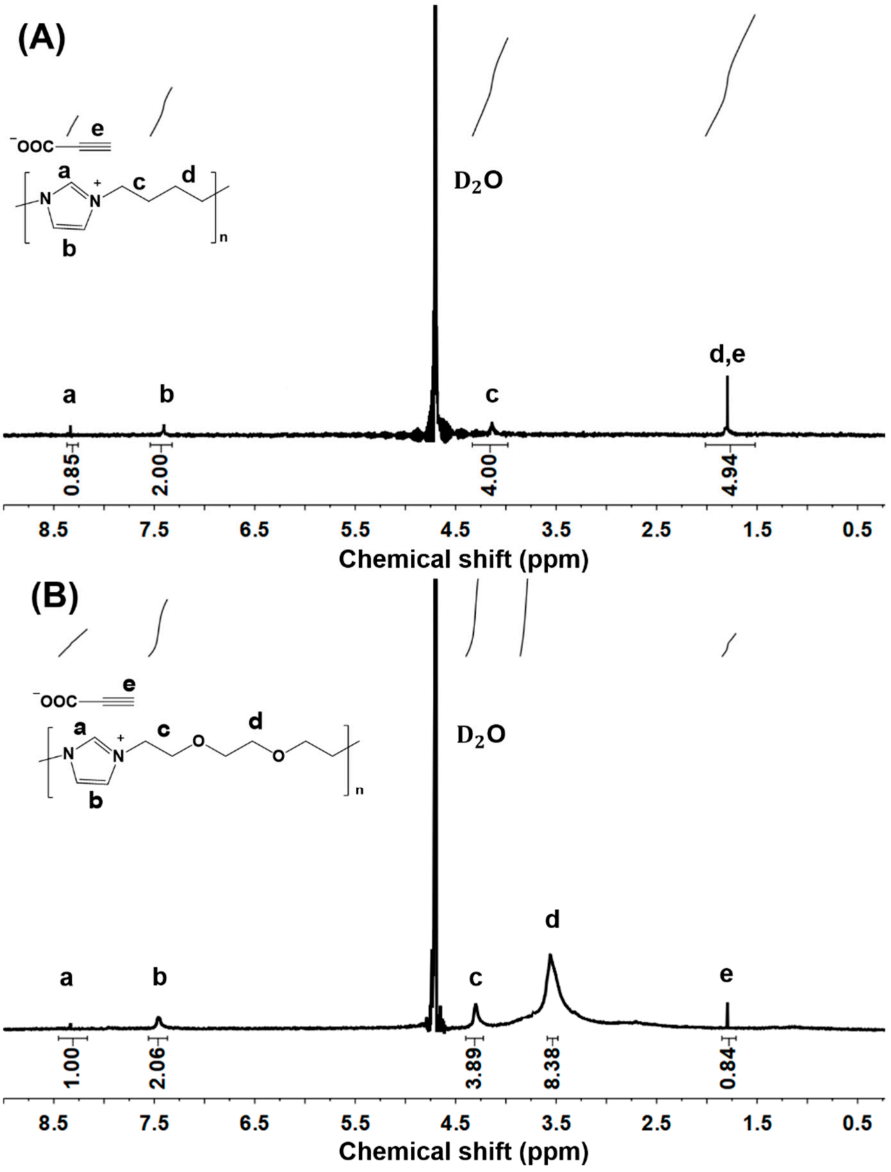

Figure S1. ^{1}H NMR (500 MHz, D_2O) of (**A**) PBP a 8.3 (d, –N–C*H*=CN$^+$) b 7.48 (d, N–C*H*=C*H*–N$^+$), c 4.25 (d, N$^+$–C*H*$_2$–C*H*$_2$–CH$_2$–CH$_2$), d,e 1.74 (s, COOH–C≡C*H*), 1.75 (m, N$^+$–CH$_2$–CH$_2$–C*H*$_2$–C*H*$_2$) and (**B**) PDP a 8.3 (d, –N–C*H*=CN$^+$), b 7.48 (d, N–CH=CH–N$^+$), c 4.25 (dd, N$^+$–C*H*$_2$–C*H*$_2$-O), d 3.55–3.75 (m, CH$_2$–O–C*H*$_2$–C*H*$_2$–O–C*H*$_2$–C*H*$_2$) and e 1.74 (s, COOH–C≡C*H*).

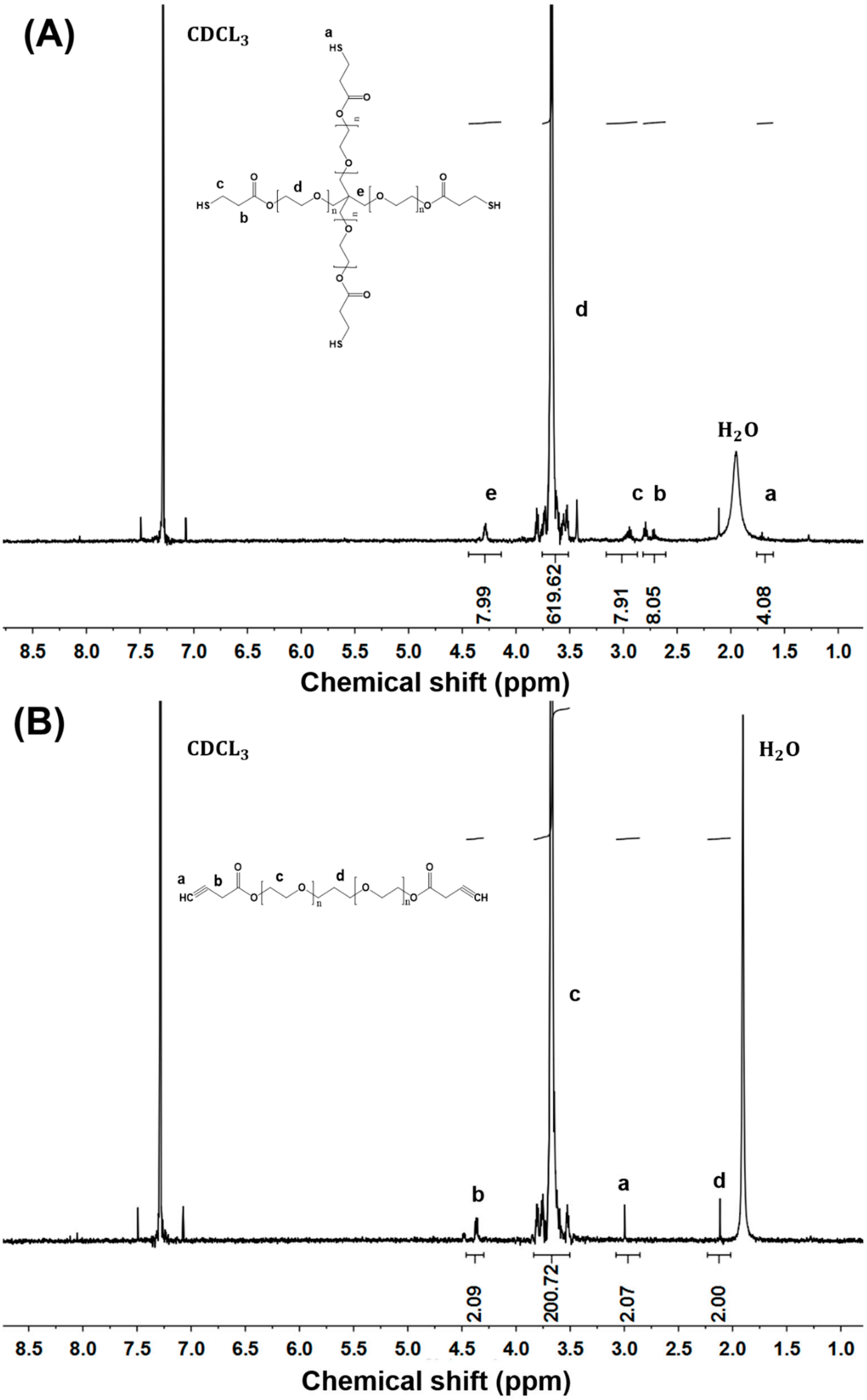

Figure S2. ^{1}H NMR (500 MHz, $CDCl_3$) of (**A**) 4 arm PEG-SH a 1.70 (s, S-*H*), b 2.66 (s, O=C–CH_2), c 2.85 (d, CH_2–SH), d 3.57–3.70 (m, CH_2–CH_2–O), e 4.26 (s, O-CH_2)) and (**B**) PEG-Alkynyl a 2.12 (s, CH_2–CH_2–CH_2), b 3.01 (s, C≡C*H*), c 3.57–3.70 (m, CH_2–CH_2–O), d 4.26 (s, CH_2–C=O).

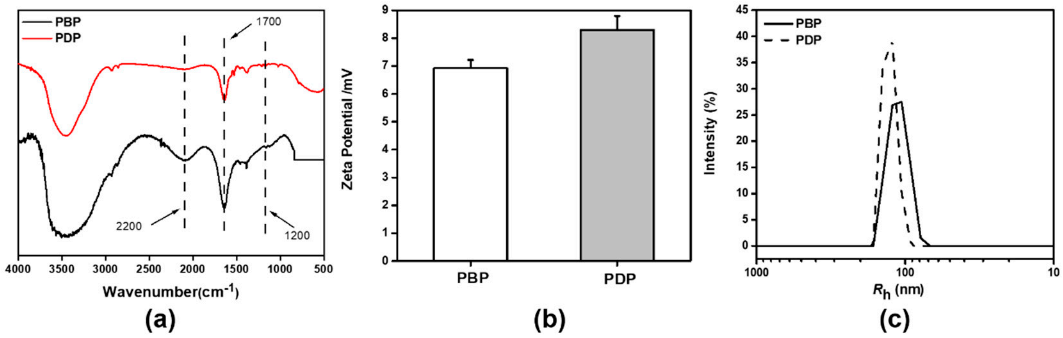

Figure S3. Characterization of poly(ionic liquids) (**a**) FTIR of PBP and PDP; (**b**) zeta potential of PBP and PDP and (**c**) nano particle size of PBP and PDP.

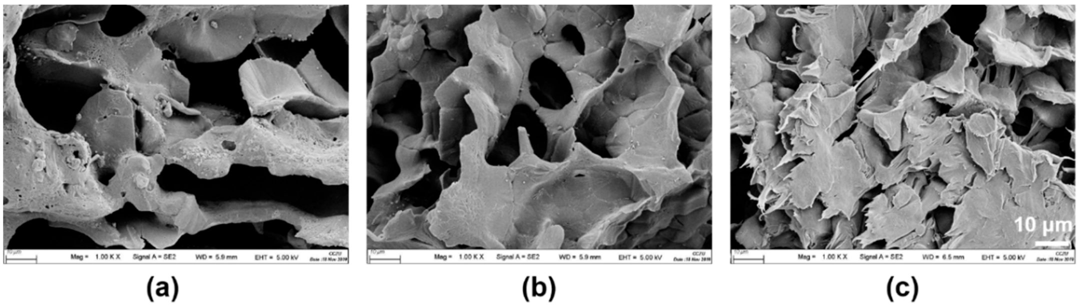

Figure S4. Surface morphology of (**a**) HN; (**b**) HB; (**c**) HD by FE-SEM; the scale bars are 10 μm.

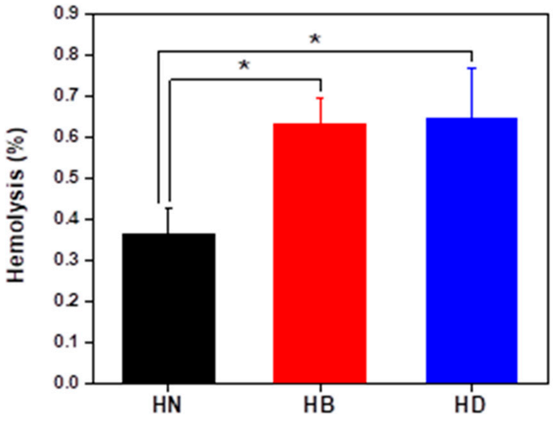

Figure S5. Hemolysis of hydrogels (n = 4, * means $P \leq 0.5$).

Table S1. Swelling characteristics and crosslinking density of injectable hydrogels in PBS (pH = 7.4) at 37 °C.

Samples	Swelling Ration (%)	Crosslinking Density (mol/cm³)
HN	754	0.13
HB	421	0.41
HD	373	0.52

Table S2. Antibacterial and cell viability of PBP and PDP.

**Samples**	**MIC (μg/mL)**		**Cell Viability (%)**
	E. coli	***S. aureus***	
PBP	250	125	90.2
PDP	62.5	25	91.3
